# Supplementary material for: Multiple mini-interviews as admission process: A study on perception of health science students in Universiti Brunei Darussalam
Source: PLoS One. 2024 Dec 17;19(12):e0314778. doi: 10.1371/journal.pone.0314778 (PMC11651595; doi:10.1371/journal.pone.0314778)
Supplement: Attachment — Submitted filename: Response to Reviewers.docx [file pone.0314778.s001.docx]

**Comments to the Author**
**Reviewer 1**

**Comment:**

The paper would benefit if the authors provided a list of objectives.

**Response:**

Objectives have been added at the end of the introduction section. (Page 6, Lines107 – 112)

**Comment:**

Although a complex study was well designed, it suffered from a poor response, which brought the numbers to a lower threshold. Nevertheless, it does show the strengths and weaknesses of the MMI.

The authors should provide more information in the study on “preparing for the MMI”. The following questions need clarification.

Did the applicants have the specific stations that they encountered in the MMI or were they given a generic set of questions?

**Response:**

They were given a generic set of questions. In the MMI, students were tested in ten individual stations that assessed the following skills:

A: Students’ motivation, qualities and achievements, communication, and self-reflection

B: Knowledge of Health Care and Current Issues

C: Ethics

D: Critical Thinking

**Comment:**

How far in advance did they receive the stations?

**Response**:

They were briefed by giving a set of instructions and information on the themes of the stations via email 2 weeks before the MMI.

They were given 5 minutes to familiarise themselves with the stations before each station began.

**Comment:**

Were they instructed on limitations in discussing their stations with fellow applicants or others?

**Response:**

The applicants were instructed not to discuss the stations with their peers.

**Comment:**

Did all applicants have access to the same quality of libraries?

**Response:**

Applicants came from different prior institutions, and therefore quality of libraries would differ.

Applicants' preparations for the MMI were dependent on their ability to prepare from various sources and not restricted to any libraries. General recommendations for the MMI preparation were made without giving specific access to libraries.

**Comment:**

The authors noted that, depending on preparation for the MMI some applicants were more comfortable with stations than other participants in the study. Was the degree of comfort with a specific station a predictor of performance?

**Response:**

Rather than comfort, MMI assessors provided a score for the global rating of the applicant during the interview. This score was based on the assessor's overall impression of the candidate, specifically looking at their communication skills and how articulated their responses were, how confident and composed they were, their professional appearance and demeanour, as well as motivation and enthusiasm during their time spent in each station, and scored separately by respective assessors pertinent to that station.

**Comment:**

Was the degree of comfort with the entire process a predictor of performance? Again, this would be useful information for planners of an MMI.

**Response:**

In our Institute's interview process, the global rating was not factored in the total scoring of applicants' performance, but rather as a deciding factor when evaluating borderline applicants (for example if two or more applicants had the same scores, the global rating will assist in making the final decision between the two).

The detailed process of MMI at PAPRSB Institute of Health Sciences UBD has been previously published separately.

Citation:

Alam F, Lim YC, Chaw LL, Idris F, Kok KYY. Multiple mini-interviews is a predictor of students' academic achievements in early undergraduate medical years: a retrospective study. BMC Med Educ. 2023 Mar 27;23(1):187. doi: 10.1186/s12909-023-04183-7. PMID: 36973779; PMCID: PMC10044430.
