# Supplementary material for: Multiple mini-interviews as admission process: A study on perception of health science students in Universiti Brunei Darussalam
Source: PLoS One. 2024 Dec 17;19(12):e0314778. doi: 10.1371/journal.pone.0314778 (PMC11651595; doi:10.1371/journal.pone.0314778)
Supplement: Attachment — Submitted filename: Response to Reviewers.docx [file pone.0314778.s002.docx]

Date:	May 06 2024 12:57AM

Subject: PLOS ONE Decision: Revision required [PONE-D-23-20665R1]

**EDITOR COMMENT 1:**

As observed in the referee evaluations of the article, providing more detailed explanations, especially regarding the analysis of qualitative data, is important for the reliability and validity of the research. The use of quantitative and qualitative data together requires evaluation with mixed research methodology. It would be appropriate to consider the discussion and conclusion sections within this framework. I believe that it would be appropriate to revise and edit the article for these reasons.
 **Response:**

Thank you for your valuable input and we agree with the comment. This paper has utilised mixed methods approach, and has interpretation of the findings are that of complementary of each method, following through in the discussion and conclusion. Please find the revised manuscript submission. Thank you for your kind reconsideration and we hope to receive your favourable decision soon.

**REVIEWER # 2 COMMENTS:**
All the reviewer's comments have been addressed satisfactorily. There is good improvement in this revised paper.

**Response:**

Thank you for your valuable comment, we greatly appreciate it.

**REVIEWER # 3 COMMENTS:
Comment #1:**

The statistical data analysis used in this paper is wrong and not suitable for the data.

**Response:**

Thank you for your valuable comment. We would appreciate further clarification on this point. Currently, we are comparing a categorical factor towards a categorical outcome, and the chi-square test is appropriately used in this univariable inference. Due to the limitation of a small sample size, higher order analysis was not possible and this is acknowledged.

**Comment #2:**

APA was not followed in the paper.

**Response:**

PLOS One requirement is Vancouver and not APA style. Thus, APA has not been followed in the manuscript.

**Comment #3:**

The discussion is poorly written and does not address the previous research.

**Response:**

We have revised the discussion section as per the comment.

Kindly find our revised manuscript submission.
