# Supplementary material for: Multiple mini-interviews as admission process: A study on perception of health science students in Universiti Brunei Darussalam
Source: PLoS One. 2024 Dec 17;19(12):e0314778. doi: 10.1371/journal.pone.0314778 (PMC11651595; doi:10.1371/journal.pone.0314778)
Supplement: Attachment — Submitted filename: Response to Reviewers.docx [file pone.0314778.s003.docx]

Date:	Oct 14 2024 05:18AM

Subject: PLOS ONE Decision: Revision required [PONE-D-23-20665R2]

**EDITOR COMMENT 1:**

In addition to the minor suggestion made by Reviewer #4 below, please also ensure that:

- APA formatting has been fully followed throughout the paper

- the discussion sufficiently incorporates previous supporting literature.
 **Response:**

APA formatting has been fully followed throughout the paper .

**REVIEWER # 2 COMMENTS:**
All the reviewer's comments have been addressed satisfactorily. There is good improvement in this revised paper.

**Response:**

Thank you for your valuable comment, we greatly appreciate it.

**REVIEWER # 3 COMMENTS:**
No Response

**REVIEWER # 4 COMMENTS:**

Although this study is not the first in its field, it is well organized and completed in a way that includes elements which can provide ideas for the future. In addition, the authors have revised the text by taking into account the suggestions of previous reviewers. It is seen that these revisions are sufficient.
Only the table number given in lines 239-240 is incorrect. It is the table where the quotations are given and it is called Table 4, but it should be Table 5.

**Response:**

The table number has been edited.
